# Assessment of Mandibular Molar Root Morphology Using Cone‑Beam Computed Tomography in Periodontitis Patients From Eastern Province: A Cross-Sectional Study

**DOI:** 10.7759/cureus.20804

**Published:** 2021-12-29

**Authors:** M Madi, Ahmed Elakel, Nourhan Aly, Roba Al Mansour, Abdullah Al Mansour, Osama Zakaria

**Affiliations:** 1 Department of Preventive Dental Sciences, College of Dentistry, Imam Abdulrahman Bin Faisal University, Dammam, SAU; 2 Department of Pediatric Dentistry and Dental Public Health, Alexandria University, Alexandria, EGY; 3 Department of Biomedical Dental Sciences, College of Dentistry, Imam Abdulrahman Bin Faisal University, Dammam, SAU

**Keywords:** molar morphology, root canal anatomy, endodontics, cone-beam computed tomography (cbct), bone loss, periodontal disease

## Abstract

Background

The aim of this study was to assess the root morphology of mandibular molar teeth using cone‑beam computed tomography (CBCT) in patients with periodontal disease.

Methods

In total, 88 patients were included in this study (70 patients with periodontitis and 18 patients with non-periodontitis). This cross-sectional study involved CBCT images taken for patients who visited the dental clinic of Imam Abdulrahman Bin Faisal University (IAU) from January 2019 to March 2021. The following data were analyzed on the mandibular molars: root length, number of root canals, root trunk, distance between roots, accessory canals in the furcation area (ACF), bone loss, and furcation involvement.

Results

The mesial root was longer than the distal root in the mandibular molars of periodontitis and non-periodontitis patients. A statistically significant difference was observed between non-periodontitis and periodontitis patients regarding the number of mesial root canals of the mandibular first molar; 70% had two root canals in non-periodontitis patients, compared with 86.1% in periodontitis patients (p = 0.04). First molars of non-periodontitis patients had significantly longer root trunks than periodontitis patients (4.65 ± 0.90 compared with 4.09 ± 1.02, p = 0.007). There was a statistically significant difference in bone loss between non-periodontitis and periodontitis patients (0% and 25% for first molars, and 2.8% and 23.6% for second molars, respectively). Accessory furcation canals were 2.9 % in second molars and 7.1 % in first molars in periodontitis patients, which were higher compared with non-periodontitis patients.

Conclusions

The first molar showed a longer root trunk in non-periodontitis patients than in periodontitis patients. The mean mesial and distal root lengths were also greater in the first than the second molar. Accessory canals in the furcation area were more observed in first molars than in second molars in periodontitis patients compared with non-periodontitis patients.

## Introduction

Periodontitis is a chronic multifactorial inflammatory disease that is associated with dysbiotic dental plaque biofilms and characterized by progressive destruction of the tooth-supporting apparatus [1,2]. In multirooted teeth, the progression of periodontal disease is usually higher in furcation areas. Thus, understanding molar root anatomy is essential for proper diagnostic and therapeutic decisions. Factors such as root trunk length, furcation entrance, root separation, and root surface area can affect diagnosis and consequently the choice of the appropriate therapy for furcally involved molars [3,4].

A bidirectional relationship exists between periodontal and endodontic problems as there are different communication pathways between the pulp and the periodontium, including accessory canals, dentinal tubules, and apical foramina [2,5]. Accessory canals have been mainly observed at the apical third of the root and in the furcation area [6-8]. These pathways for communication result in pathological microorganism migration between the periodontium and the dental pulp, which can lead to endo-periodontal lesions [9].

Morphological variations in multirooted teeth increase the risk of furcation involvement and bone loss by the persistence of periodontal disease that creates a challenge in therapeutic decisions [10].

It is known that endodontic disease can also provoke furcation involvement in the so-called primary endodontic lesion [11]. The term endo-periodontal lesion describes a pathologic communication between the pulpal and periodontal tissues of a given tooth. Endo-periodontal lesions are classified according to their signs and symptoms that directly affect the treatment and prognosis of the tooth, depending on the extension and severity of the periodontal disease [12].

Having adequate knowledge about the morphological presentation of the roots and furcation of periodontally involved teeth is essential for better clinical practice, involving diagnosis, prevention, and treatment of periodontal disease. Thus, the aim of this study was to assess the root and canal morphology in mandibular molar teeth using cone‑beam computed tomography (CBCT) in patients with periodontal disease.

## Materials and methods

This cross-sectional study involved CBCT images taken for patients who visited the dental clinic from January 2019 to March 2021. Ethical approval was obtained from the institutional review board (IRB 2020-02-110). The CBCT scans were taken using the standard protocol for diagnosis and measurement, and written informed consent for participation was taken. Initially, a total of 88 scans were collected. The current study was conducted in accordance with the Strengthening the Reporting of Observational Studies in Epidemiology (STROBE) guidelines [13].

The inclusion criteria were as follows: patients who visited the dental clinics seeking dental treatment and in whom CBCT scans were performed and CBCT images of good quality and showing both arches. The exclusion criteria were as follows: patients with incomplete facial growth (less than 18 years old), patients with systemic disease, bone pathology or taking medications that affect bone turnover, incomplete patient records, partial CBCT scans, and presence of radiographic artifacts.

All scans were taken using the KODAK 9500 Cone Beam 3D System (Carestream, Rochester, NY, USA) with a flat panel detector (imaging area was a cylinder of 15-20.6 cm height and 9-18 cm diameter). Standard resolution mode (voxel size of 0.2 mm) was selected (standard exposure parameters were set to 90 kV tube voltage, 10 mA tube current, and 10.8 seconds exposure time). The examination was performed by 360° rotation in the occlusal position with the patient standing and closing their jaws. The CS 3D Imaging Software (3.4.3. Carestream Health Inc., Atlanta, USA) was used for the evaluation of the CBCT obtained DICOM, and the full volumes were assessed. The software provides panoramic, axial, and cross-sectional sagittal images on the same screen for mandibular molar measurement.

Radiographic assessment

The following parameters were measured on the right and left mandibular molars.

Panoramic View of the CBCT

1. Root length, which represented the distance from the cementoenamel junction to the root apex.

2. Root trunk length, which is the distance from the cementoenamel junction to the furcation fornix (the roof of the furcation).

3. Distance between mesial and distal roots.

4. Bone loss of mandibular molars (average of both mesial and distal sides).

5. Furcation bone loss.

Coronal View of the CBCT

1. Number of canals in mesial and distal roots.

2. Furcation bone loss.

Sagittal View of the CBCT

1. Number of accessory canals in the furcation area (ACF).

2. Number of canals in mesial and distal roots.

Statistical analysis

Inter‑examiner reliability was determined by measuring all parameters on 20 mandibular first molars. The kappa coefficient between the two examiners was 0.9, which reflects an overall excellent agreement.

Data were analyzed using IBM SPSS for Windows (version 23.0), and significance was inferred at p-value < 0.05. Normality was checked for all variables using descriptive statistics, plots, and normality tests. Means and standard deviation (SD) were calculated for quantitative variables, while frequencies and percentages were calculated for qualitative variables. Comparisons between gingivitis and periodontitis cases were done using independent sample t-test for quantitative variables and Fisher’s exact and chi-square tests with Monte Carlo correction (whenever indicated) for qualitative variables.

## Results

**Figure 1:**
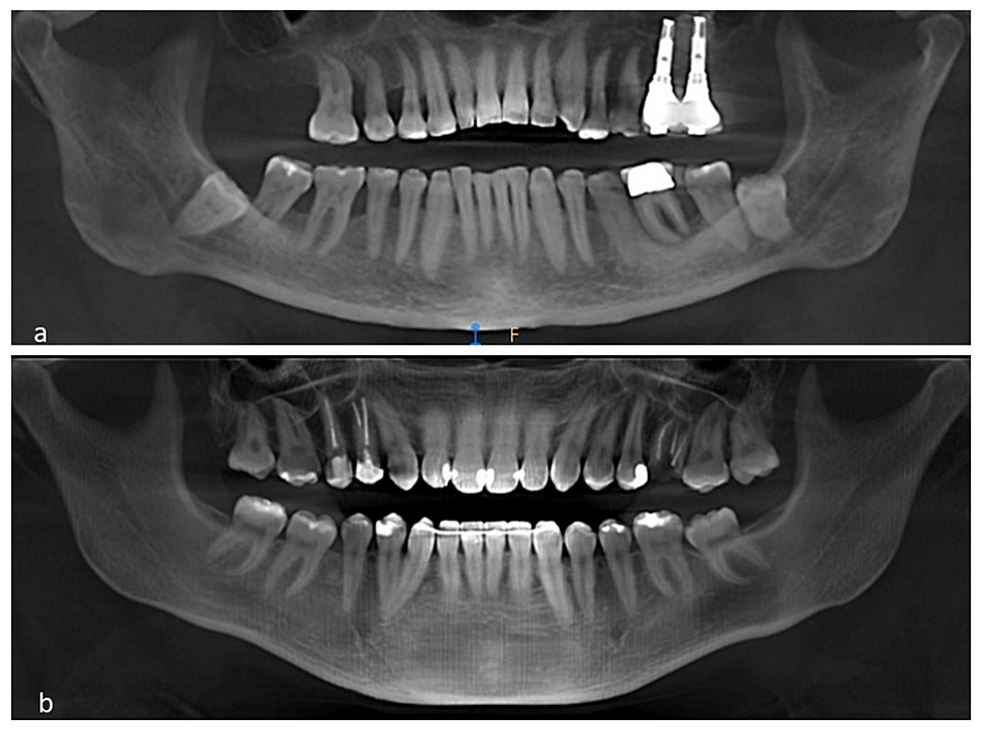
Panoramic view of the cone-beam computed tomography images a: Periodontitis case. b: Non-periodontitis case

In total, 88 patients were included in this study with 352 examined teeth (70 periodontitis patients (280 teeth) and 18 non-periodontitis patients (72 teeth) (Figure 1).

Of these participants, 52 were females and 36 were males, with mean age ± SD of 29.79 ± 9.53 (Table 1).

**Table 1:** Demographic characteristics of the study participants

		Gingivitis (n = 18)	Periodontitis (n = 70)	Total (n = 88)	P-value
Age (mean ± SD)		27.00 ± 0.00	30.00 ± 9.88	29.79 ± 9.53	0.78
Gender (n (%))	Male	8 (44.4%)	28 (40%)	36 (40.9%)	0.73
	Female	10 (55.6%)	42 (60%)	52 (59.1%)	

The main morphological characteristics of mandibular first and second molars are presented in Table 2 and Table 3.

**Table 2:** Morphological characteristics of mandibular first molars (#18 and #30) n: number of patients, N: number of teeth, PMC: Monte Carlo-corrected p-value, PFE: Fisher’s exact test was used *Statistically significant at p-value < 0.05

		Non-periodontitis (n = 18) (N = 30)	Periodontitis (n = 70) (N = 122)	Total (n = 88) (N = 152)	P-value
Mesial root length (mm) (mean ± SD)		15.52 ± 2.66	15.47 ± 2.68	15.48 ± 2.67	0.92
Distal root length (mm) (mean ± SD)		13.94 ± 2.48	13.86 ± 2.87	13.87 ± 2.79	0.88
Number of canals (n (%))	Two canals	9 (30%)	17 (13.9%)	26 (17.1%)	P_MC_: 0.11
	Three canals	19 (63.3%)	94 (77%)	113 (74.3%)	
	Four canals	2 (6.7%)	11 (9%)	13 (8.6%)	
Number of mesial canals (n (%))	One canal	9 (30%)	17 (13.9%)	26 (17.1%)	0.04*
	Two canals	21 (70%)	105 (86.1%)	126 (82.9%)	
Number of distal canals (n (%))	One canal	28 (93.3%)	111 (91%)	139 (91.4%)	P_FE_: 1.00
	Two canals	2 (6.7%)	11 (9%)	13 (8.6%)	
Distance to furcation (mm) (mean ± SD)		4.65 ± 0.90	4.09 ± 1.02	4.20 ± 1.02	0.007*
ACF (n (%))		1 (2.8%)	10 (7.1%)	11 (6.3%)	P_FE_: 0.67
Distance between roots (mm) (mean ± SD)		2.18 ± 0.90	2.34 ± 0.96	2.31 ± 0.95	0.42
Bone loss (mean ± SD, n (%))		0 (0%)	2.35± 0.9, 35 (25%)	2.35 ± 0.9, 35 (19.9%)	P_FE_: 0.001*
Furcation bone loss (n (%))		0 (0%)	11 (7.9%)	11 (6.3%)	P_FE_: 0.19

**Table 3:** Morphological characteristics of mandibular second molars (#19 and #31) n: number of patients, N: number of teeth, PMC: Monte Carlo-corrected p-value, PFE: Fisher’s exact test was used *Statistically significant at p-value < 0.05

		Non-periodontitis (n = 18) (N = 29)	Periodontitis (n = 70) (N = 120)	Total (n = 88) (N = 149)	P-value
Mesial root length (mm) (mean ± SD)		15.44 ± 2.65	15.07 ± 2.48	15.14 ± 2.51	0.47
Distal root length (mm) (mean ± SD)		14.40 ± 2.72	13.76 ± 2.54	13.89 ± 2.58	0.24
Number of canals (n (%))	Two canals	0 (0%)	5 (4.2%)	5 (3.4%)	P_MC_: 0.36
	Three canals	25 (86.2%)	105 (87.5%)	130 (87.2%)	
	Four canals	4 (13.8%)	10 (8.3%)	14 (9.4%)	
Number of mesial canals (n (%))	One canal	0 (0%)	5 (4.2%)	5 (3.4%)	P_FE_: 0.58
	Two canals	29 (100%)	115 (95.8%)	144 (96.6%)	
Number of distal canals (n (%))	One canal	25 (86.2%)	110 (91.7%)	135 (90.6%)	P_FE_: 0.48
	Two canals	4 (13.8%)	10 (8.3%)	14 (9.4%)	
Distance to furcation (mm) (mean ± SD)		4.48 ± 0.95	4.17 ± 0.95	4.23 ± 0.95	0.12
ACF (n (%))		0 (0%)	4 (2.9%)	4 (2.3%)	P_FE_: 0.68
Distance between roots (mm) (mean ± SD)		2.28 ± 0.86	2.13 ± 0.97	2.16 ± 0.95	0.43
Bone loss (mean ± SD, n (%))		1 (2.8%)	2.52± 1.19, 33 (23.6%)	2.52± 1.19, 34 (19.3%)	P_FE_: 0.009*
Furcation bone loss (n (%))		0 (0%)	8 (5.7%)	8 (4.5%)	P_FE_: 0.28

For non-periodontitis and periodontitis patients, the mean length of the mesial root was about 15.52 and 15.47 mm, respectively, compared with 13.94 and 13.86 mm distal root length. Most of the included molars had three root canals (63.3% and 77% in the case of first molars, and 86.2% and 87.5% in the case of second molars for non-periodontitis and periodontitis patients, respectively). There was a statistically significant difference in the number of mesial root canals of the first molar between non-periodontitis and periodontitis patients; 70% had two root canals in non-periodontitis patients compared with 86.1% in periodontitis patients (p = 0.04). First molars of non-periodontitis patients had significantly greater distances to furcation than periodontitis patients (4.65 ± 0.90 compared with 4.09 ± 1.02, p = 0.007). There was a statistically significant difference between non-periodontitis and periodontitis patients in bone loss, where the loss was higher in the case of periodontitis (25% compared with 0%, p = 0.001, and 23.6% compared with 2.8%, p = 0.009, in the case of the first and second molars, respectively). The mean radiographic bone loss observed in periodontitis patients for mandibular first and second molars were 2.35 ± 0.9 and 2.52 ± 1.19, respectively.

**Figure 2:**
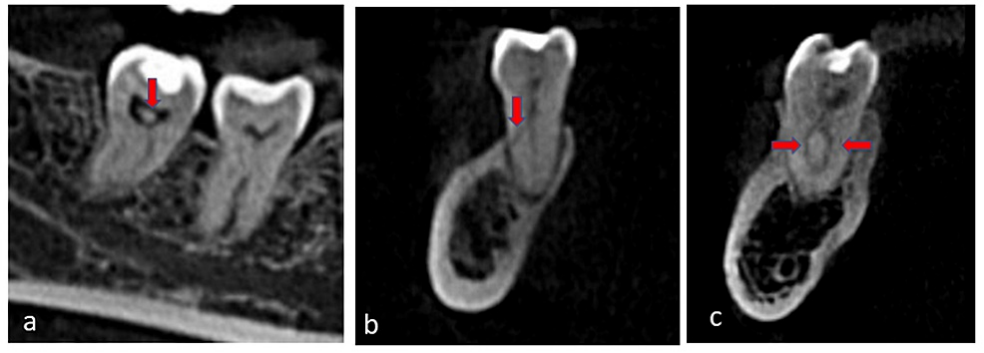
Cone-beam computed tomography images a: Pulp stone in the pulp chamber of the right second mandibular molar, sagittal view (arrow). b: Lateral canal in the distal root of the left second mandibular molar, coronal view (arrow). c: Canal morphology in the mesial root of the right mandibular first molar, coronal view (arrow)

Only six (3.4%) molars had pulp stones: five (3.6%) in periodontitis patients and one (2.8%) in non-periodontitis patients (Figure 2). For periodontitis and non-periodontitis patients, the accessory canals in the furcation (ACF) were 2.9 % in second molars and 7.1 % in first molars, and about 0% in second molars and 2.8% in first molars, respectively.

## Discussion

Our findings showed a relation between the morphological characteristics of mandibular molars and periodontal disease. There was a statistically significant difference between periodontitis and non-periodontitis patients regarding the number of mesial root canals of the mandibular first molar. First molars of non-periodontitis patients had significantly longer root trunks than that of periodontitis patients. There was a statistically significant difference in bone loss between non-periodontitis and periodontitis patients. This morphological anatomical variation can increase the risk of periodontal disease progression, and this would affect disease prognosis influencing therapeutic decision-making. Previous studies [4,14] described that shape, length, and distance between molar roots are important parameters in tooth prognosis, as they can affect the stability of molars.

Matherne et al. [15] and Mittal et al. [16] showed the superiority of axial images of CBCT over other diagnostic methods in locating additional canals. Thus, CBCT imaging is not only noninvasive but also a highly sensitive method for morphological studies.

A previous study that was conducted on 251 mandibular molars extracted from Saudi patients found that 94% had two roots and 6% had three roots, while 42% of the teeth had three canals and 58% had four canals [17]. This is in agreement with our findings, where 63.3% of mandibular first molars in non-periodontitis and 77% in periodontitis patients had three root canals.

Ross et al. [18] examined 340 maxillary and mandibular molars radiographically in 170 patients with periodontitis. They demonstrated that 29% of molars had fused roots. However, they did not investigate the morphological aspects of fused roots in detail. Thus, a detailed examination of the morphological abnormalities that could be present in roots lost due to periodontitis is essential for a better understanding of the factors that would affect disease progression [19].

Al-Qudah et al. [20] reported that the majority of mandibular first molars had three (48%) or four (46%) canals. Of the distal roots, 54% had one canal and 45% had two canals, and mandibular second molars showed the most distal roots (79%) and the two rooted molars had only one canal. Of the mesial roots, 81% had two canals. Our findings, on the other hand, showed that, for mandibular first molars, 86.1% had two mesial root canals and 91% had one distal root canal. For mandibular second molars, 95.8% had two mesial canals and 91.7% had one distal canal.

Our results showed that the furcation distance significantly contributed to the pathogenesis of periodontitis. First molars of non-periodontitis patients had significantly greater distances to furcation than periodontitis patients. This is in agreement with a previous study [21] that reported that the root trunk of the first molar was greater than the second and third molars with an average distance of 2.6 mm, making it less prone to periodontal diseases.

Anatomical variations of the furcation area may contribute to the initiation and persistence of periodontal disease. Haznedaroglu et al. [22] showed that patent furcal accessory canals were detected in 24% of mandibular first molars and 20% of mandibular second molars out of 200 molar teeth extracted; however, the cause of extraction was not investigated in this study. This was consistent with our study, which showed that the accessory canals in the furcation (ACF) were 2.9 % in second molars and 7.1 % in first molars, and about 0% in second molars and 2.8% in first molars for periodontitis and non- periodontitis patients, respectively. According to previous studies, root trunk dimensions play an important role in the periodontal disease process due to their significant relation to both prognosis and treatment of the tooth [5,23].

In our study, first molars of non-periodontitis patients had significantly longer root trunks than periodontitis patients (4.65 ± 0.90 compared with 4.09 ± 1.02, p = 0.007), making them less vulnerable to extensive furcation involvement. Mandelaris et al. [24] reported that the mean buccal root trunk lengths of 134 extracted human mandibular molars were 3.14 mm, irrespective of their periodontal condition. They used a high magnification microscope with digital software to measure precisely morphological features in the furcation area rather than visual measurements and calipers.

Vertical attachment loss that extends to the furcation area results in the loss of one-third of the total periodontal support of the tooth. Thus, a molar with a short root trunk is more vulnerable to furcal involvement [25]. Similarly, in the current study, the mandibular first molar showed more favorable morphological features regarding the root trunk length than the second molar.

The limitations of this study are smaller sample size, lack of different ethnicity subjects, and lack of correlation between the clinical and radiographic findings.

## Conclusions

In this study, the first molars showed longer root trunk length (4.65 ± 0.90) in non-periodontitis than periodontitis patients (4.09 ± 1.02). The mean mesial and distal root lengths were also greater in the first than the second molar. Accessory canals in the furcation area were observed more in first molars (7.1 %) than in second molars (2.9 %) in periodontitis patients. Thus, morphological variations in multirooted teeth especially at the furcation area have a great influence on periodontal disease progression and furcation involvement.
